# 503 Navigating Recovery While Being Unhoused: Understanding Burn and Cold Injury Rehabilitation Journeys

**DOI:** 10.1093/jbcr/iraf019.132

**Published:** 2025-04-01

**Authors:** Carly Marincasiu, Caitlin Orton, Maiya Pacleb, Geun-woo Oh, Deja Nicholas, Leslie Enzian, Barclay Stewart

**Affiliations:** Harborview Medical Center, University of Washington; University of Washington; University of Washington Medicine Regional Burn Center; Harborview Medical Center, University of Washington; Harborview Medical Center, University of Washington; Harborview Medical Center, University of Washington; University of Washington

## Abstract

**Introduction:**

People experiencing homelessness (PEH) face a higher risk of burn and cold injuries due to poverty, reliance on makeshift cooking and warming devices, and limited access to safety promotion activities. Additionally, PEH experience more severe injuries and complicated recovery journeys, compounded by disabilities, mental illness, and barriers to accessing rehabilitation services. Therefore, we aimed to identify specific rehabilitation goals, needs, and experiences of PEH with a burn or cold injury and how to facilitate access to services needed for long-term, successful community reintegration.

**Methods:**

We interviewed 10 PEH living with burn or cold injuries. Seven participants were less than six months from the date of injury, one participant was 11 months from injury, and two participants were over one year from injury at the time of their interview. We leveraged the Levesque framework for healthcare access to better understand rehabilitation goals and assess how PEH prefer to engage with healthcare following their injuries. Interview recordings were transcribed, and two coders used inductive and deductive approaches for analysis using grounded theory.

**Results:**

Participants reported a range of experiences related to their rehabilitation journeys and related strategies for improving acceptability and effectiveness of rehabilitation services. We identified four key themes: (1) PEH have unique rehabilitation goals—develop person-specific rehabilitation plans contextualized to the living circumstances of PEH; (2) Rehabilitation occurs in the context of additional complex health needs—the negative impacts of burn and cold injuries are further aggravated by sub optimally managed acute and chronic health conditions and poor social conditions that require co-management for successful rehabilitation; (3) PEH often have complicated relationships with healthcare systems—integrate trauma-informed care, harm reduction approaches, case management, and peer navigation to strengthen trust between PEH and healthcare services; and (4) Competing health and non-health services—improve access and utilization of health and non-health services by co-locating services where able, supporting communication and transportation, and including rehabilitation liaisons into homelessness community services.

**Conclusions:**

PEH emphasized their specific needs for rehabilitation goals, services, and opportunities to strengthen both health and non-health service delivery.

**Applicability of Research to Practice:**

Incorporating PEH and their lived experiences into rehabilitation services can improve their acceptability and effectiveness. Suggested strategies for doing so include the development of tailored rehabilitation goals, case management, peer navigation, communication and transportation support, and coordination between health and non-health services.

**Funding for the Study:**

The contents of this abstract were developed under a grant from the National Institute on Disability, Independent Living, and Rehabilitation Research (NIDILRR grant #90DPBU0005). NIDILRR is a center within the Administration for Community Living (ACL), Department of Health and Human Services (HHS). The contents of this abstract do not necessarily represent the policies of NIDILRR, ACL, HHS, and do not assume endorsement by the Federal Government.

